# From the eyes and the heart: a novel eye-gaze metric that predicts video preferences of a large audience

**DOI:** 10.3389/fpsyg.2015.00579

**Published:** 2015-05-12

**Authors:** Christoforos Christoforou, Spyros Christou-Champi, Fofi Constantinidou, Maria Theodorou

**Affiliations:** ^1^Center for Applied Neuroscience, University of CyprusNicosia, Cyprus; ^2^Research and Development Division, R.K.I Leaders Ltd.Larnaca, Cyprus; ^3^Department of Psychology, University of CyprusNicosia, Cyprus

**Keywords:** eye-tracking, neruromarketing, neurocinematics, heart rate variability, video evaluation

## Abstract

Eye-tracking has been extensively used to quantify audience preferences in the context of marketing and advertising research, primarily in methodologies involving static images or stimuli (i.e., advertising, shelf testing, and website usability). However, these methodologies do not generalize to narrative-based video stimuli where a specific storyline is meant to be communicated to the audience. In this paper, a novel metric based on eye-gaze dispersion (both within and across viewings) that quantifies the impact of narrative-based video stimuli to the preferences of large audiences is presented. The metric is validated in predicting the performance of video advertisements aired during the 2014 Super Bowl final. In particular, the metric is shown to explain 70% of the variance in likeability scores of the 2014 Super Bowl ads as measured by the USA TODAY Ad-Meter. In addition, by comparing the proposed metric with Heart Rate Variability (HRV) indices, we have associated the metric with biological processes relating to attention allocation. The underlying idea behind the proposed metric suggests a shift in perspective when it comes to evaluating narrative-based video stimuli. In particular, it suggests that audience preferences on video are modulated by the level of viewers lack of attention allocation. The proposed metric can be calculated on any narrative-based video stimuli (i.e., movie, narrative content, emotional content, etc.), and thus has the potential to facilitate the use of such stimuli in several contexts: prediction of audience preferences of movies, quantitative assessment of entertainment pieces, prediction of the impact of movie trailers, identification of group, and individual differences in the study of attention-deficit disorders, and the study of desensitization to media violence.

## 1. Introduction

The evaluation of video advertising aims to characterize the profile of a video advertisement and predict its potential impact on the consumer in order to select the best advertisement or advertisement storyline before it is aired. Recently, there has been a growing interest in using methods from neuroscience and experimental psychology in order to identify metrics that could explain and predict the effectiveness of video advertising (Vecchiato et al., 2011a). Moreover, such metrics could be useful in a diverse set of research studies, such as investigating gender (Vecchiato et al., 2014b) and cross-cultural (Vecchiato et al., 2014a) differences during the observation of video advertisement, the analysis of movie to inform cognitive film theory (Smith, 2013) and the study of deficient affective processing in individuals with high callous-unemotional traits (Fanti et al., 2015), among others.

Observation of the eye movements of consumers, while watching a video, provides details on what elements they are looking at, information that could ultimately determine the consumers perception of the video (Dorr et al., 2010). Eye-tracking technology provides a powerful tool which records viewers eye-movements while watching on-screen stimuli. It has been extensively used to quantify audience preferences in advertising research such as package testing (Tonkin et al., 2011), printed advertisement pretesting (O'Malley and Latimer, 2012), usability studies (Bernhaupt et al., 2007), and web-content visibility (Wang and Day, 2007). However, analysis methodologies of eye-tracking data mostly apply to static visual stimuli or stimuli where visual areas of interest can be explicitly defined. When it comes to video advertisement, the use of eye-tracking data has been limited to informational video genre where specific elements and frames of the video can be explicitly identified as areas of interest (AOI). This is primarily due to methodological challenges in identifying informative metrics on the dynamic content (Dorr et al., 2010) that can predict the post-air audience preferences of the video advertisement.

Traditionally, quantitative eye-tracking analysis methodologies for advertisement pretesting rely on defining specific visual AOI of the stimuli under study. AOI can be, for example, product or brand visuals, call-to-action elements, key visual illustrations, special offers, headline elements, slogans, or any other element controlled for performance. Subsequently, the raw gaze-information is used to calculate the relevant statistics for each area of interest, which might include the time to first fixation, observation length, number of fixations, and average fixation duration, among others. By comparing these statistics to either normative data or statistics from a different execution of the advertisement, the advertisement is identified as performing either above or below par. However, oftentimes a video advertisement needs to communicate the brands key message (or value proposition) through a storyline or script which aims to take the viewer on a narrative journey in the hope that the viewer will be able to associate the message with the brand. In storyline-based video advertising, no single frame or element can be explicitly identified as being of interest, rather the entire sequence of frames work together to drive the communication. This property of the narrative- or storyline-based video renders the application of AOI-based methodologies in this context problematic.

More recently, alternative methods for the analysis of eye-movements recorded during video viewing (Smith, 2013) have been proposed. Typically, these methods define metrics which measure the consistency of eye-movements of different observers. The proposed metrics include clustering-based methods (Goldstein et al., 2007) which measure the percentage of fixations falling within a main cluster, string editing methods (Clauss et al., 2004) where gaze paths are encoded in string representation, attentional synchrony (Smith and Henderson, 2008), correlation metrics between scan-paths (Hasson et al., 2008b), and information theoretic metrics (Rajashekar et al., 2004), among others (Dorr et al., 2010). The fundamental problem with such methods is that there is no direct (known) mapping between the eye position and its perceptual consequences (Dorr et al., 2010). Moreover, none of the metrics proposed in the literature have been shown to carry information that can predict post-air audience preferences of video advertisement.

An alternative approach to characterizing video content performance has been the use of neural activity, typically measured through electroencephalography (EEG) or functional magnetic resonance imaging (fMRI)-derived blood-oxygenation-level-dependent (BOLD) signals. Indeed, a number of methods have been proposed for deriving useful components from EEG signals which can serve as metrics for evaluating static or dynamic content. These methods typically rely on identifying spatial, temporal, or spectral projections of the raw neural signal (Christoforou et al., 2008, 2010a, 2013) which, in turn, serve as metrics of the stimulus impact on an observer. Such methods have been used in many application areas including Brain Computer Interface (Blankertz et al., 2008), Robotic-telepresence (Christoforou et al., 2010b), maximization of throughput in high-performance tasks (Parra et al., 2008), and more recently, in video analysis (Dmochowski et al., 2012; Kong et al., 2013). Similarly, methods which rely on fMRI treat the level of neural response in a fixed region as a predictive metric of behavioral measures. Indeed, inter-subject correlation (ISC) in neural activity has been linked to successful memory encoding (Hasson et al., 2008a) and has been shown to be modulated by engaging narrative stimuli (Lankinen et al., 2014), as well as scenes with high arousal and negative emotional valance (Hasson et al., 2004).

In particular, a number of approaches have been proposed that use EEG measures to characterize the effects of video advertising on consumers. For example, Vecchiato et al. (2011b) investigated the changes in EEG frontal asymmetry in alpha and theta bands, during the observation of pleasant and unpleasant video advertisements. Their analysis showed an asymmetrical increase of the theta and alpha activity in relation to the observation of pleasant (vs. unpleasant) video advertisements. Moreover, this activity is negatively correlated with the degree of pleasantness perceived by the participants. In an alternative approach, Kong et al. (2012) investigated the memorization variation during the observation of video advertisements. In particular, the authors proposed a metric, which depends of the overall power in the theta band in EEG signals, as an index of memorization. They suggest that their metric reflects the memorization level during observation of video advertisements. In subsequent work, Kong et al. (2013) proposed the impression index which combines both the memorization and attention index during consumers observing video advertisement while tracking their EEG activity. They suggest that the proposed index tracks variations of the cerebral activity related to cognitive tasks, such as observing video advertisements, and helps to judge whether scenes in the video advertisement are impressive or not. Typically, the majority of these EEG metrics (which rely on EEG frontal asymmetry) are shown to be modulated during the observation of video advertisement, and to be related with indirect or subjective measures of cognitive abilities or emotional traits (i.e., pleasant, unpleasant, attention, memorability etc.) as expressed by a small sample of participants. However, to the best of our knowledge, none of the proposed metrics have been shown to predict direct measures of post-air performance of video advertisement on large audience.

Recently, neural reliability a metric of the overall power of ISC components extracted from EEG has been used as an independent variable to predict viewers preferences in video advertisements (Dmochowski et al., 2014). In particular, Dmochowski et al. (2014) used ads aired during the 2012 Super Bowl, and looked at how synchrony in EEG outcomes predicted audience preferences measured by a massive online survey. Subsequently, they proposed that the resulting EEG metric might be a marker of engagement. In this paper, we build on the approach and ideas in Dmochowski et al. (2014) and propose a novel metric based only on eye-tracking data that predicts viewers preferences in video advertisements.

Specifically, motivated by evidence relating ISC measures of neural activity to the expected engagement of viewers (Dmochowski et al., 2012) to the video stimuli, and the use of neural reliability a marker derived from ISC as a predictor of post-air performance of video advertisement (Dmochowski et al., 2014), we suspected that group measures of lack-of-attention could be captured by eye-tracking data and could serve as suitable predictors of the post-air performance of video advertisements. Specifically, we hypothesize that (a) eye-tracking measures can be used to define a robust metric of divergence of viewers attention, (b) such a metric can be calculated from a small number of viewers, and (c) the metric can predict post-air audience preferences of narrative-based videos.

Thus, in the present study, we define a novel metric from eye-tracking data collected from viewers while watching narrative-based advertisement. Unlike traditional eye-tracking analysis methods, the proposed metric does not rely on defining any specific AOI in video scenes, which enables it to be directly applied to storyline-based videos. Moreover, the metrics calculation only uses gaze-location information (i.e., the x- and y-coordinates), which can be robustly measured by any off-the-shelf eye-tracking system; hence, the metric calculation does not require any specialized eye-tracking measures such as saccades, micro-saccades, or pupil dilation, which are only available on high-end (and expensive) eye-tracking systems. These features make the proposed metric easy to calculate, affordable, and scalable. Moreover, we explore the relation between the proposed metric and the heart-rate variability indices. Finally, to evaluate the predictive power of the resulting metric, we report results on the post-air performance of video advertisements telecasted during the 2014 Super Bowl.

## 2. Materials and methods

### 2.1. Experimental paradigm

#### 2.1.1. Participants

A total of 16 participants (11 female, 5 male) were recruited with self-reported normal or corrected-to-normal vision and participated in the study. All participants were fluent in the English language. The minimum, median, and maximum ages of the participants were 19, 22, and 24, respectively. Participants were compensated for their participation.

#### 2.1.2. Procedure

Each participant was seated in a comfortable chair and briefed on the objective of the study. In particular, participants were informed that they would be shown a set of video advertisement and movie trailers and that they would have to report their opinion of each video using an on-screen questionnaire. Following a short preparation and calibration session (see Section 2.3), each participant was presented with 13 video advertisements (12 of which were advertisements aired during the Super Bowl 2014 telecast, and one video advertisement was an animatic, see Section 2.2). Following each advertisement viewing, a questionnaire would pop up on the screen asking the participant to report the degree to which they liked the advertisement, and whether they would consider sharing a link of the video on their Facebook account. Following the display of all 13 video advertisement, the participants were showen 5 movie trailers of current movies. Subsequently, the participants were shown all 13 video advertisements for a second time. For stimulus presentation, we used the open source software OpenSesame (Matht et al., 2012). The videos were shown at a frame rate of 23 Hz and an aspect ratio of 4:3, with the sound on (on-monitor speakers were used to deliver the sound). The resolution of the stimulus presentation monitor was set to 1024 × 758 px for all video advertisement. The order of the video advertisement was randomized across participants, but the order was preserved for each participant for the two viewings. The Cyprus Bioethics Committee approved all the study procedures, and a consent form was obtained from all the study participants.

### 2.2. Video dataset and post-air performance scores

The Super Bowl final telecast provides a convenient framework to evaluate the predictive utility of the proposed metric. It has been the most watched televised event in the United Stated for the last 5 years and reaches an audience of 110 million viewers. Traditionally, the advertisements are debuted on the day of the Super Bowl and are typically onetime-only advertisements. The investment for producing and airing a video advertisement during the telecast is typically large reportedly 3 million USD for airing a 30-s spot. Hence, the video advertisements aired during the Super Bowl telecast are very well-executed and often deliver the brands message through well-designed storylines. In addition, the video advertisements are evaluated on post-air performance that is made publicly available. For our study, we selected 12 advertisements that communicated the brands message through a story and were aired during the 2014 Super Bowl telecast. As a post-air performance score for the selected videos, we used the USA TODAY Super Bowl Ad-Meter Score (AD-Meter, 2014) which is a measure of the likeability of the advertisement. The Ad-Meter is a survey conducted by the USA TODAY as a live poll during the telecast of the game. The survey is conducted annually since 1989 and has become the standard in advertisement performance of Super Bowl video advertisements. Moreover, the survey results carry a value on their own as the winning video advertisements enjoy additional exposure from media coverage. The videos selected for this study and their corresponding Ad-Meter Scores are listed in Table 1.

**Table 1:** **Video advertisement aired during 2014 Super Bowl telecast and their corresponding Ad-Meter Rating**.

**ID**	**Description (Advertiser)**	**Ad-Meter rating**
1	Grace (Cheerios)	6.75
2	Puppet Master (Go Daddy)	4.66
3	Hero's Welcome (Budweiser)^*^	7.21
4	Time Machine (Doritos)	7.13
5	100,000 Miles (Volkswagen)^*^	6.24
6	Nice (Hyundai)	6.1
7	Puppy Love (Budweiser)^*^	8.29
8	Romance (Chevrolet)^*^	6.18
9	Cowboy Kid (Doritos)	7.58
10	Bodybuilder (Go Daddy)	4.04
11	Sixth Sense (Hyundai)	6.87
12	Luxury (KIA)^*^	6.09

The ID was used as identifier of each video in the results section. The duration of the videos were either 30 s or 60 s (superscript

* in the videos description column identifies videos with 60 s duration.)

### 2.3. Data collection and preprocessing

The current study is part of a broader project which aims to investigate the neurophysiological modulations from multiple modalities during video viewing. For that purpose, during the data collection process, the data was collected from eye-tracking, heart-rate sensors, and EEG signals. The EEG data collected was not utilized for the current study; however, for a complete description of the experimental apparatus, we briefly discuss their data collection process. The EEG data collection was performed using the Neuroscience Platform-as-a-Service (R.K.I. Leaders, Cyprus) which employed a BioSemi Active-two system (BioSemi, Amsterdam, Netherlands) at a sampling rate of 512 Hz. Subjects were fitted with a standard 32-electrode cap following the international 10/20 system. The preparation procedure took about 10 min during which all electrodes were placed and the impedance of all sensors was kept below 20 *k*Ω. EEG data was collected for the entire duration of the experiment. The EEG data preprocessing and data analysis are outside the scope of this study.

For the purpose of this study, the gaze data was collected during the entire experiment. We employed an eye-tracking unit, a presentation unit, and an analysis unit. Gaze data was sampled at 60 Hz, and the spatial accuracy was kept within 0.5°. The eye-tracking unit was placed in front of the participant and below the stimulus monitor, with the camera-to-eye distance at about 60 cm. Prior to data collection, a 9-point calibration session was executed to ensure a correct mapping of the gaze data points and screen coordination. In addition, event-markers were sent to the gaze stream to allow synchronization of the video frames and the gaze data. The gaze-coordinate stream was epoched between −1000 ms before each videos start time and 1000 ms after the videos finish time. Each gaze point was then re-referenced to the videos start time. Gaze streams for all participants and viewings were then used to calculate the *eye-gaze Divergence Index* (*iGDI*) metric as described in Section 2.4. The analysis was performed using a custom Matlab code (Mathworks Inc.) (MATLAB, 2010).

HRV was collected throughout the experiment using photoplethysmography (PPG) signal sensors attached to the left ear lobe. Relevant research has shown that the peaks of the PPG signal correspond to the R-peaks (heartbeats) obtained using electrocardiographic (ECG) equipment, suggesting that the PPG is a valid measure of cardiac activity including HRV measures in both healthy people and people suffering from cardiovascular diseases (Nitzan et al., 1998; Murthy et al., 2001). The output of the PPG sensor was digitally sampled, and an online Butterworth low pass filter, with a cutoff at 7 Hz, was employed to remove any high-frequency noise present in the signal. Artifact preprocessing was conducted on the inter-beat interval (IBI) data following the guidelines in Goedhart et al. (2007). Data sets were visually inspected for abnormal fluctuations using Kubios software (Biosignal Analysis and Medical Imaging Group, 2008). HRV was measured as the root mean square of successive differences (RMSSD) in the IBIs (Camm et al., 1996). To establish baseline HRV, the participants were instructed to sit comfortably and breathe normally for 5 min while no stimulus was presented on the screen. HRV responses obtained during the video presentation were adjusted for baseline HRV via the computation of percentage change (Stern et al., 2001). Change scores were computed by subtracting the baseline from HRV during the video viewing. The primary reason for adjusting the HRV responses for baseline was to remove concomitant variation in the response and thus improve the precision of comparisons (Keiser, 1989). The computation of percentage change allowed us to normalize the data before subsequent statistical analysis (Hancock et al., 1988).

### 2.4. Eye-gaze divergence index (iGDI)—definition

In this section, we provide the details of the calculation of the *iGDI* metric which has been specifically designed to quantify the dispersion in gaze positions between two different renditions of a video advertisement. First, we define a metric of the dispersion between two viewings of a video segment.

Let **V** ∈ ℝ^2 × *N*^ and **W** ∈ ℝ^2 × *N*^ be matrices representing two sequences of gaze positions.

Denote **v**(*n*) ∈ ℝ^2^ and **w**(*n*) ∈ ℝ^2^ as the *n*th columns of matrices **V** and **W**, respectively, each representing a gaze position, and let *v*_*i*_(*n*), *w*_*i*_(*n*), *i* ∈ {1, 2} correspond to the *i*th elements of vectors **v**(*n*) and **w**(*n*), respectively. The first element corresponds to the x-coordinate of the gaze point, while the second element is the y-coordinate of the gaze point.

We define the dispersion score between the two renditions as the average pairwise Euclidean distance between **V** and **W**. Formally,

(1) $$D(V,W)=\frac{1}{N^{2}}\sum_{n_{1}}^{N}\sum_{n_{2}}^{N}\sqrt{\sum_{i\;=\;1}^{2}(v_{i}(n_{1})-w_{i}(n_{2}){)}^{2}}$$

#### 2.4.1. Between-viewings dispersion scores

During our experiment, we had multiple participants watching a video advertisement twice. Let **V**^(1)^, **V**^(2)^, …, **V**^(*s*)^, …, **V**^(*S*)^ where **V**^(*s*)^ ∈ ℝ^2 × *N*^ represent the gaze path of participant *s* during a short video segment on the first viewing of the video. Similarly, let **W**^(1)^, **W**^(2)^, …, **W**^(*s*)^, … **W**^(*S*)^ where **W**^(*s*)^ ∈ ℝ^2 × *N*^ represent the gaze path of participant *s* during the same short video segment, but on the second viewing of the video. For the purpose of our analysis, we consider the dispersion score between the two viewings and define the renditions for two video segments as follows:

(2) $$V=[V^{\left(1\right)},V^{\left(2\right)},\ldots ,V^{\left(s\right)},\ldots V^{\left(S\right)}]$$

(3) $$W=[W^{\left(1\right)},W^{\left(2\right)},\ldots ,W^{\left(s\right)},\ldots W^{\left(S\right)}]$$

We calculate the dispersion between these two renditions on the matrices **V** and **W** defined in Equation (2) using Equation (1). We refer to this score as the between-viewings dispersion score. Note that the number of columns in **V** and **W** are now *S* × *N*. Also note that *N* is selected to correspond to a small time window of a video segment (for our study we use windows of 250 ms). We compute the dispersion scores for the entire video in a time-resolved fashion by employing a sliding window with 250 ms duration and a shift of the window occurring every 50 ms (80% overlap between successive windows).

#### 2.4.2. Within-viewings dispersion scores

Similarly, we define the within-viewings dispersion scores as follows:

(4) $$D_{within}(\bar{V})=\frac{1}{N^{2}}\sum_{n_{1}}^{N}\sum_{n_{2}}^{N}\delta_{n_{1},n_{2}}\sqrt{\sum_{i\;=\;1}^{2}({\bar{v}}_{i}(n_{1})-{\bar{v}}_{i}(n_{2}){)}^{2}}$$

where δ_*ij*_ = 1 iff *i* ≠ *j*. We note that the within-viewings dispersion score depends on gaze paths of a single video viewing. For the purposes of this study, we calculate the within-viewings dispersion scores for the first, and second viewings of the video [i.e., *D*_*within*_(**V**) and *D*_*within*_(**W**)].

The use of short video segments in the calculation of the dispersion scores is motivated by the observation that during a segment participants are exposed to exactly the same frame sequence that aims to guide their attention to a particular storyline, and thus a similar gaze path. The degree to which the video sequence achieves this objective is reflected by the dispersion score. Scenes for which the viewers focus their attention in nearby locations in the video sequence should have a smaller dispersion score, while scenes that do not manage to guide viewers attention are expected to have a larger dispersion score. We hypothesize that the scenes with extreme dispersion scores carry information about the inability of the video to keep users engaged, which in turn reflects in the post-air performance of the video. In the following paragraph we present to the method for categorizing such extreme scores.

#### 2.4.3. Scenes with extreme dispersion scores

Here we provide the details of classifying dispersion scores for each window as being either divergent (i.e., has extreme dispersion value) or non-divergent. The underlying principle here is to model the probability distribution of the dispersion scores under a null hypothesis that the video frames do not guide viewers attention according to the storyline or in any systematic pattern. To estimate the probability under null hypothesis, we use Monte-Carlo sampling to generate a histogram of the distribution and use it to fit a normal distribution. Each sample is drawn by first randomizing the matrices **V** and **W** across different (random) time windows of the same video and second by calculating its dispersion score using Equation (1). The mean value of the fitted distribution *D*_*null*_ serves as a threshold for classifying the windows. Formally, a window is labeled as divergent based on the following decision rule:

(5) $$F(D(V,W))\{\begin{array}{l} 1\text{ if }D(V,W)\geq {\bar{D}}_{null}\\ 0\text{ otherwise} \end{array}$$

where *F*(*D*(**V**, **W**)) = 1 denotes a window which is to be labeled as divergent and *F*(*D*(**V**, **W**)) = 0 denotes a window which is to be labeled as non-divergent. Note that the decision rule (Equation 5) is applicable for both with-viewing and between-viewings dispersion scores [i.e., by replacing *D*(**V**, **W**) with the appropriate within-viewings dispersion score].

#### 2.4.4. Eye-gaze divergence index

The *iGDI* metric for a particular video is then defined as the fraction of divergent windows. Formally, let **d** = [*D*_1_, *D*_2_, …, *D*_*T*_]^⊤^ be a vector with the dispersion scores (either within-viewings or between-viewing) at different consecutive windows spanning the length of the video and denote **f** = [*F*(*D*_1_), *F*(*D*_2_), …, *F*(*D*_*T*_)]^⊤^ as the binary vector categorizing the windows as divergent and non-divergent, then the *iGDI* is defined as

(6) $$iGDI:=\frac{1}{T}d^{⊤}f$$

For our analysis, we calculate the *iGDI* metric for both between- and within-viewings. In reporting the results, we reserve the symbol *iGDI* without any subscript to refer to the between-viewings *iGDI* score, and use the notation *iGDI*_*v*1_ and *iGDI*_*v*2_ to refer to the within-viewings *iGDI* score, for the first and second video viewings, respectively.

#### 2.4.5. Frame-by-frame insights on video advertisement

The model provides information about which scenes contribute either positively or negatively to the *iGDI* score. Such information is useful in identifying individual scenes in the advertisement that do not perform at par and taking corrective action (i.e., removing or replacing them) to improve the videos overall *iGDI* score. In addition, the visual inspection of attention maps during those scenes can provide further information on which specific elements of the scene most likely contribute to the large dispersion scores and thus provide insights into taking corrective measures before airing the advertisement. Moreover, we consider the level of agreement between the two within-viewings models in identifying similar frames as being distracting. In particular, we compare the number of windows during each video for which the two models agree in identifying a scene as being divergent.

### 2.5. Regression model on iGDI to ad-meter valuation indexes

To evaluate the ability of the proposed *iGDI* metric to predict post-air audience preferences of each video advertisement, we fitted a univariate regression model, where the *iGDI* metric serves as the independent variable and the Ad-Meter score as the dependent variable. Formally, the model is defined as:

(7) $$y_{admeter}=b_{1}\;iGDI+b_{0}$$

where the *y*_*admenter*_ denotes the predicted Ad-Meter score, *iGDI* is the proposed metric, and *b*_0_, *b*_1_ denote the intercept and slope parameters of the regression model. To train the model, we first calculated the dispersion score for each video at multiple time windows using the Equations (1) or (4). The time interval of each window was 250 ms, and subsequent windows overlapped by 200 ms (i.e., the window was shifted by 50 ms). The sequence of all the time windows spans the entire duration of the video advertisements. Second, each window was classified as being either divergent or non-divergent using the decision rule of Equation (5). Finally, the *iGDI* metric for each video presented was calculated using Equation (6). The regression model was fitted using least-squares criterion. A separate prediction model was fitted for the *iGDI* calculated on the between-viewings dispersion scores and for the *iGDI* metrics calculated on the within-viewings dispersion scores. Moreover, in the case of within-viewings dispersion scores calculated using Equations (4, 6) the model was trained on two separate datasets: one dataset using the gaze data recorded during the first viewing of the video and another one using gaze data recorded during the second viewing of the video.

#### 2.5.1. Generalizability of prediction model

To obtain an estimate of the generalizability of the regression model on new videos, we employed a leave-one-out cross validation procedure. In particular, the model was fitted/trained *N* times, where the *iGDI* scores of (N–1) videos were used to fit the model, and the one excluded from the training procedure was used as a test case to calculate the predicted score. The predicted values for the *N* videos were then compared to the true Ad-Meter scores. Performance of the model was reported in terms of the variance explained and Mean squared Error (MSE) on both the fitted model and the predictive model.

### 2.6. Heart rate variability index

In the present study, we assessed the relationship between the proposed *iGDI* metric measuring the divergence of eye-gaze patterns across subjects and HRV indices that are sensitive to attention allocation levels. The selection of HRV was motivated by its application as a marker of cardiac function which is modulated by activity changes in the Parasympathetic (PNS) and sympathetic (SNS) divisions of the Autonomous Nervous System (ANS). In particular, activity in the PNS division of the ANS (via vagal verve innervations at the sinus node level) decreases the heart rate, while activity in the SNS division contributes to the increase in the heart rate. In essence, HRV refers to the degree to which the time interval between successive heart beats fluctuates as a result of the influences of the SNS and PNS. This is of particular importance as previous relevant research has reported cardiac deceleration, and hence increase in HRV indices relating to PNS, with increased attention allocation both in adults and infants (Richards and Casey, 1991; Tripathi et al., 2003; Thomas et al., 2012).

There are different methods to define heart-rate variability, which include Standard Deviation between the NN^1^ (SDNN), the fraction of consecutive NN intervals that differ by more than 50 ms (pNN50), and the Root Mean Square of Successive Differences (RMSSD) (Camm et al., 1996). In the current study we used the RMSSD index for HRV because of its statistical properties as explained in Camm et al. (1996), and its relation with measures reflecting high frequency components of the respiratory range indicating increased parasympathetic influences to the heart and thus cardiac deceleration. Formally, the RMSSD score is calculated as follows:

(8) $$RMSSD(r)=\sqrt{\frac{1}{I-1}\sum_{i\;=\;1}^{I-1}((r(i)-r(i+1){)}^{2})}$$

where **r** ∈ ℝ^*I*^ is a vector of *I* consecutive IBIs^2^ measured during the period of a video viewing. The RMSSD score, calculated over the entire duration of the each video, was then adjusted for the baseline HRV via the computation of percentage change which allowed us to normalize the data before subsequent analysis.

To investigate the relation between the proposed metric and the level of attention allocation of participants during video viewing, we used the RMSSD metric calculated from the HRV of the group of participants viewing each video advertisement. As there was no significant difference in the RMSSD between the first and second viewing, *t*_(11)_ = 0.38, *p* > 0.05, we combined these data in the following analysis. In addition, a set of *Shapiro*−*Wilktests*(*W*) were conducted to test whether dependent variables deviated from normality. The examination of the normality of the distribution of cardiac RMSSD data (*W* = 0.96, *p* > 0.05) and of the *iGDI* metric (*W* = 0.97, *p* > 0.05) suggested that the obtained data was normally distributed. Therefore, a Pearson product-moment correlation coefficient was computed to assess the relationship between the % change in RMSSD and the eye-tracking metric measuring the divergence of eye-gaze patterns across subjects.

## 3. Results

### 3.1. Between-viewings prediction model results

**Figure 1:**
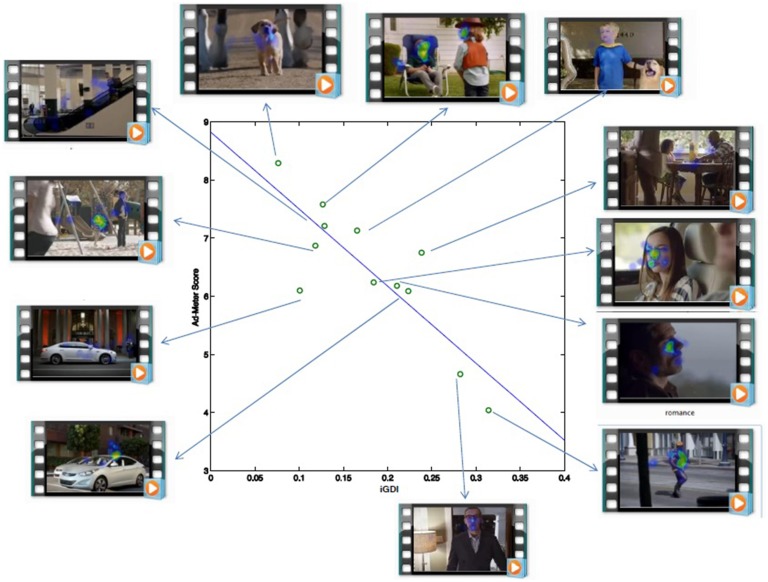
**Eye-gaze Divergence (between-viewings) calculated on small sample is predictive of preference ratings in large audience**. Vertical axis: subjective ratings for 2014 Super Bowl advertisements collected from a large online audience (USA Today Ad-Meter). Horizontal axis: *iGDI* metric across 10 subjects during each advertisement. Solid line indicates the linear prediction of population ratings from *iGDI*: 70% of variance in population ratings is explained. (*R* = 0.84, *R*^2^ = 0.70, *F*_(1, 10)_ = 23:4, *p* < 0.0006). Video advertisement pictures (left side of scatter plot, top-to-bottom): Heros welcome, Sixth Sense, Luxury, Nice. Video advertisement pictures (top side of scatterplot, left-to-right): Puppy Love, Cowboy Kid, Time Machine. Video advertisement pictures: (right side of scatterplot, top-to-bottom): Grace, 100,000 Miles, Romance, Bodybuilder. Video advertisement pictures: (right side of scatterplot): Puppet Master.

Results of the between-viewings regression indicate that the *iGDI* explained 70% of the variance (*R* = 0.84, *R*^2^ = 0.701, *F*_(1, 10)_ = 23.4, *p* < 0.0006). Figure 1 shows the scatterplot and the linear predictive model which shows the negative correlation between the *iGDI* and the Ad-Meter scores; as the Ad-Meter score decreases, the *iGDI* increases, and vice versa. This negative correlation is as was expected because the Ad-Meter measures a positive attribute of the video (i.e., overall impact) whereas the *iGDI* was designed to capture a negative attribute of the video (i.e., its inability guide viewers attention).

Results on the generalizability of the between-viewings regression model show a small Root Mean Square Error (*RMSE* = 0.77) and Mean Absolute Percentage Error (*MAPE* = 10%). In addition, the cross validation coefficients across models show little variance across the different trained models (*std*(β_1_) = 1.1, *std*(β_0_) = 0.20) suggesting the stability of the regression-line parameters (slope and intercept). Moreover, the cross-validation coefficient is *R*^2^_*cv*_ = 0.53 and the cross-validation shrinkage is *Shrinkage* = 0.17.

### 3.2. Within-viewings prediction model results

**Figure 2:**
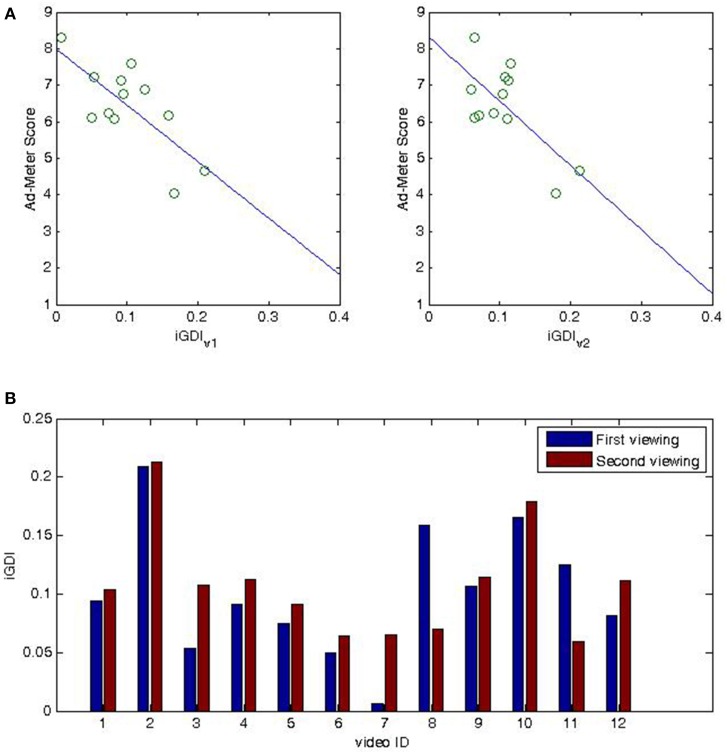
**Eye-gaze Divergence (within-viewing) calculated on small sample is predictive of preference ratings in large audience**. Vertical axis: subjective ratings for 2014 SuperBowl advertisements collected from a large online audience (USA Today Ad-Meter). **(A)** Horizontal axis: *iGDI*_*v*_1__ (left-top figure) and *iGDI*_*v*_2__ (right-top figure) metrics across 10 subjects during the first and second viewing of each advertisement, respectively. Solid line indicates the linear prediction of population ratings from *iGDI*_*v*_1__ and *iGDI*_*v*_2__, respectively: 54% of variance in population ratings is explained from *iGDI*_*v*_1__ (*R* = 0.73, *R*^2^ = 0.538, *F*_(1, 10)_ = 11.67, *p* < 0.006); 48% of variance in population ratings is explained from *iGDI*_*v*_2__ (*R* = 0.69, *R*^2^ = 0.482, *F*_(1, 10)_ = 11.67, *p* < 0.01). **(B)** (bottom-figure) modulation of *iGDI*_*v*_1__ and from *iGDI*_*v*_2__ for each video; horizontal axis correspond to video id as listed in Table 1.

Results of the within-viewings regression model indicate that the *iGDI*_*v*1_ explained 54% of the variance (*R* = 0.73, *R*^2^ = 0.5385, *F*_(1, 10)_ = 11.6708, *p* < 0.0066), while *iGDI*_*v*2_ explained 48% of the variance (*R* = 0.69, *R*^2^ = 0.4828, *F*_(1, 10)_ = 9.3339, *p* < 0.0121). Figure 2 shows the scatter-plot and the linear prediction line for the two models. As in the case of between-viewings, there is a negative correlation between the *iGDI* and the Ad-Meter scores. We further evaluated the predictive power of these two models on novel data using the leave-one-out cross-validation procedure outlined above. Both models show a small prediction error with *iGDI*_*v*1_: *RMSE* = 0.82, *MAPE* = 13%, *iGDI*_*v*2_: *RMSE* = 0.94, *MAPE* = 14%. The modulation of the *iGDI* metric of each video across the two viewings are shown in Figure 2 (*bottom row*).

### 3.3. Frame-by-frame insights on video advertisement

**Figure 3:**
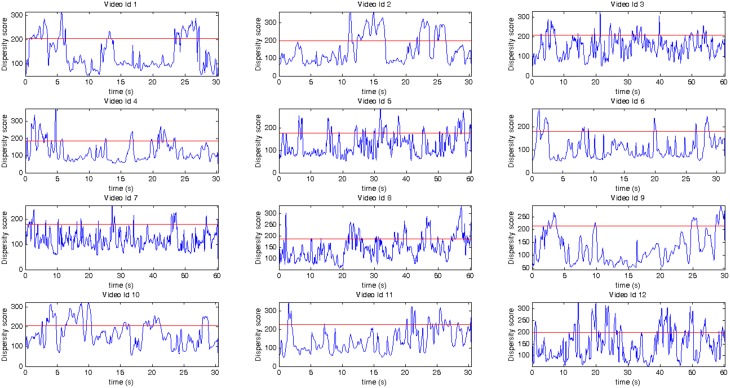
**Each plot corresponds to the Dispersion Score for each video across time, video id correspond to those listed in 1**. Vertical axis: dispersion scores calculated across the two viewings of a video. Horizontal axis: Time in seconds for each video. The red horizontal line corresponds to the disparity score decision rule boundary (i.e., time intervals whose dispersion scores above the red line are classified as distracting).

Figure 3 shows the modulation of dispersion scores for the entire duration of each video separately. The red line denotes the boundary for the decision rule defined in Equation (5); thus, the scenes with dispersion scores above the red line are considered divergent and increase the *iGDI* score of the video. It is interesting to note that the videos with the lowest Ad-Meter score (i.e., video-id 2 and video-id 10) show extended consecutive time points of distracting frames, which correspond to the frames that the video creator should visually inspect for improvement. Moreover, results on comparing the agreement between the two within-viewings models show that the classification decision of the two models agree in 88% of the time-windows considered (mean overlap proportion = 0.88 ± 0.0379, min = 0.82, max = 0.95).

### 3.4. Heart-rate variability results

The Pearson product-moment correlation coefficient was computed to assess the relationship between the RMSSD % change and the eye-tracking metric measuring the divergence of eye-gaze patterns across subjects. Results show a moderate negative relationship between RMSSD % change and the *iGDI* metric, *r*_(22)_ = −0.33, *p* < 0.05.

## 4. Discussion

In this paper we have presented a novel metric to quantify the degree of divergence of viewers' attention, measured by the divergence of eye-gaze patterns across subjects, when presented with a narrative- or storyline-based video, and have shown that the metric carries predictive information about the post-air audience preferences about the video. The proposed metric, which is calculated from eye-tracking data of a small sample of individuals, is shown to greatly predict the preferences of the larger audience viewing the video during a telecast. To evaluate the predictive power of the proposed metric, we used advertisements aired during the 2014 Super Bowl final. For post-air performance score, we used the audience preferences measured by the USA TODAY Ad-Meter Score. Two distinct versions of the metric have been proposed and evaluated: one using gaze data between two viewings of a video (between-viewings metric) and one using gaze data within a single viewing (within-viewings metric), as described in the Materials and Methods Section. The results show that both instantiations greatly predict the post-air performance of video advertisements; however, the metric calculated with the data between the two viewings explains more of the variance in the dependent variable and with smaller generalization error. This suggests that the ability of the video to not lose viewers attention after multiple repetitions encodes additional information for predicting a videos post-air performance. Indeed, by investigating the within-viewings metric for the first and second view, we have shown the modulations of the metric calculated within the first and second viewings separately. Moreover, we have shown that the proposed metric provides frame-by-frame details of those time windows of the video that contribute positively (or negatively) to the predicted performance. One can pinpoint those scenes of the video which need to be edited to improve its post-air performance and thus our metric is a powerful tool for optimizing video performance before airing them. Finally, we investigated the relation between the proposed metric and the level of attention allocation of participants during viewing. The results show a moderate negative relationship between Heart Rate Variability index (RMSSD) and the proposed metric. These results suggest that increases in RMSSD (which likely indicate cardiac deceleration) are related to decreases in the divergence of eye-gaze patterns across subjects as measured by the proposed metric.

Our approach differs from traditional eye-tracking methodologies which require specific AOI to be explicitly defined for the key frames of the video. A key advantage of the proposed metric is that it is calculated using only the gaze-coordinate data obtained from off-the-shelf eye-tracking systems and does not require the defining any AOI. This is of particular importance in the study of narrative-based videos because for this video-genre no single frame or element can be explicitly identified as being of interest, but rather the entire sequence of frames work together to drive the communication. Moreover, to the best of our knowledge, this is the first time that an eye-tracking based metric has been shown to carry predictive information that can directly link the metric to the post-air preferences of a large population.

Our findings show the highly predictive power of our proposed metric which is comparable to the state-of-the-art methods which use high-end and expensive neuroimaging modalities such as fMRI and EEG. In particular, our metric achieves prediction accuracy equivalent to a recently published method that uses EEG and fMRI based ISC (Dmochowski et al., 2014) on a similar video dataset (i.e., Super Bowl ads). Moreover, our approach differs from that in Dmochowski et al. (2014) in terms of the video attributes measured. In particular, the EEG/fMRI ISC metric is shown to capture the degree of engagement (i.e., the proportion of scenes the viewers are particularly engaged with in the video), whereas in our approach the proposed eye-tracking metric captures degrees of extreme divergence of attention. Note that the two metrics are not absolute opposites (i.e., engagement is not equivalent to lack of attention divergence); thus, they capture different information from the video.

More importantly, the use of extreme divergence of attention as a metric suggest a shift in perspective in the context of narrative-based video evaluation (and in general in dynamic stimuli evaluation). Current approaches, whether relying on neuroimaging or self-report measures, focus on positive attributes of the video stimuli such as scenes that cause maximal levels of engagement, reduce workload, or have maximal memory recall. Our findings suggest that negative attributes of the video (i.e., the level of extreme divergence of viewers attention) are at least equally important in defining the impact of the narrative videos. One may interpret these results as suggesting that the impact of the viewers preferences interplay between engaging scenes and scenes of the video stimuli associated with divergence of attention. On the one hand, engaging scenes offer the opportunity to remember specific elements of the stimuli. These elements can serve as iconic-scenes to trigger messages or brand associations. Moreover, the more engaging a scene is, the more likely the viewer will follow the videos narrative. On the other hand, scenes with extreme attention divergence captured via the proposed metric act as interest-landmines. The more the scenes in a video that allow the viewer attention to diverge from a common attention path, the more opportunities for the viewer to disconnect from the narrative and the message, and lose interest.

Given the evidence showing that both engagement (captured by ISC of neuroimaging data) and attention divergence (captured by our proposed metric via eye-tracking data) carry predictive information about the videos post-air performance, and the realization that the two metrics could carry complementary information, we hypothesize that combining the two metrics would result in even more accurate predictive power. Moreover, we hypothesize that combined metrics will carry predictive information of different video types. In future work, we plan to investigate and characterize the predictive power of combining ISC based metrics from EEG and the proposed metric on various video types including movies and other narrative-based videos.

Finally, the proposed metric can be calculated on any narrative-based video stimuli (i.e., movie, narrative content, emotional content, etc.), and thus has the potential to facilitate the use of such stimuli in several contexts. In particular, we envision that the proposed metric can be useful in the field of cognitive film theory (Smith, 2013) by informing the editing and staging process of film making and by helping understand how the visual information influence viewers perceptions. Similarly, the proposed metric could be used in predicting the impact of movie trailers and in the quantitative assessment of entertainment pieces. Moreover, the metric can be used to identify group and individual differences in clinical populations (Fanti et al., 2015) such as people with attention-deficit disorders and the study of desensitization to media violence. For example, we hypothesize that by comparing dispersion scores across multiple repetitions of violent (vs. non-violent) video stimuli, the metric can help quantify the degree of media violence desensitization.

### Conflict of interest statement

The corresponding author serves as Chief Technology Officer at R.K.I Leaders Ltd. A non-provisional patent applications was submitted under the US patent office which in part relates to the current work. The authors declare that the research was conducted in the absence of any commercial or financial relationships that could be construed as a potential conflict of interest.

## Footnotes

^1^NN intervals refers to the different between two successive heart-beats, also refer to as inter-beat intervals.

^2^In heart-rate variability literature, elements of vector **r** are referred to as R–R, N–N or IBI.
